# Symbolic annihilation of Syrian refugees by Turkish news media during the COVID-19 pandemic

**DOI:** 10.1186/s12939-021-01472-9

**Published:** 2021-06-11

**Authors:** Alev Yücel

**Affiliations:** Faculty of Communication, Department of Media, Istanbul Bilgi University, Santralistanbul Kampusu, Eski Silahtaraga Elektrik Santrali Kazim Karabekir Cad. No: 2/13 34060 Eyupsultan, Istanbul, Turkey

**Keywords:** COVID-19 pandemic, Symbolic annihilation, Syrian refugees, Turkey, Stigmatization, Media

## Abstract

**Background:**

This article presents a discussion about the representation of Syrian refugees in Turkish news media during the COVID-19 pandemic. Media play a critical role during pandemics by affecting values, opinions, public knowledge about stigmatized groups. When media ignore and stereotypically represent a social group, the social value of the group decreases, and their problems are trivialized.

**Method:**

I analyzed data provided by Media Monitoring Center which is an independent media company in Turkey. Sample was selected to reveal news representation of Syrian refugees between March 11, 2020 (the first confirmed case of COVID-19 in Turkey) and August 20, 2020 (the time of this research). Mostly watched television evening (prime) news, the most widely circulated newspapers, and the most visited online news platforms were analyzed. By using content analysis method, the news stories about Syrian refugees were examined.

**Results:**

It has been revealed that Turkish news media overwhelmingly acclaimed for Turkey’s polices on Syrian refugees and the criticized the European Union policies towards refugees in the news stories. Even though almost 4 million Syrian refugees live in Turkey, with the largest refugee population in the world, Turkish news media ignored the plight of Syrian refugees.

**Conclusion:**

Results of the study demonstrate that Syrian refugees were symbolically annihilated by Turkish news media during the COVID-19 pandemic. The concealment of information and symbolic annihilation of disadvantaged groups could potentially cause health disparities and irreparable harm to public health. Moreover, inequities exacerbate when predicaments of stigmatized groups are ignored in the news media. Further studies are needed to reveal the impact of the media on health disparities among stigmatized groups during the pandemics.

## Introduction

Coronavirus disease (COVID-19) started as an epidemic limited to China in December 2019 and spread across the countries accompanied by a rapid rise in number of cases and deaths. The World Health Organization (WHO) declared a Public Health Emergency of International Concern (PHEIC) in January 2020 and announced a pandemic on March 11, 2020 [1]. Calling on all countries to combat the spread of the disease, the WHO aimed at taking measures against severe negative impacts of the pandemic. The first case of COVID-19 was confirmed on March 11, 2020 in Turkey. It was later than all its neighbor countries except Syria ([2] p2).^1^

Considering that Syrian refugees in Turkey hold “temporary protection” status since 2014, not granting full citizenship rights, permanent residency, regular employment and even claiming to refugee status led to precarious situation.^2^ The restrictive framework of temporary protection makes them more vulnerable to abuse and exploitation ([5] p317–8).^3^ The pre-existing inequalities and impoverishment of Syrian refugees have exacerbated by the pandemic. Pandemics are out of the ordinary periods marked by high levels of uncertainty and risk, people with high perception of risk for infection tend to blame sociological outgroups for the outbreak of the disease to overcome fear when diseases are lethal. Disadvantaged groups including immigrants, ethnic, racial, religious, and sexual minorities are stigmatized as disease vectors who pose threat to health and security of the society ([8] p462). This can lead to increasing stigmatization, scapegoating, and discrimination ([9] p246–7). Stigmatization includes labeling, stereotyping, status loss and discrimination which co-occur in the power context ([10] p367). First, human differences are labeled, second labeled person is stereotypically characterized, third status loss and discrimination occur at the last stage. Being refugee is a stigma by itself, which leads to discrimination in many aspects of life [11]. Refugees are more vulnerable to stigma, hate speech and discrimination during the pandemics [12–14].

Immigrants and ethnic groups have been historically stigmatized as carriers of disease in the second half of the twentieth century [15]. People belonging to certain ethnic groups and minorities have been stigmatized for spreading the virus during the COVID-19. Stigmatization, disinformation, misinformation, and hate speech fuel the spread of the virus and discriminatory speech, which can result in discrimination against certain groups in terms of COVID-19 issues [16]. News coverage activates public knowledge and public perception is influenced more when public relies on news for public affairs information those beyond the understanding of personal experience. ([17, 18] p17). Media representations influence values, norms, beliefs, opinions by selecting some aspects of a perceived reality and making them more salient ([19] p52–3). Valued cultural groups are frequently represented in the media but unvalued groups such as refugees or outgroups have been historically marginalized, trivialized, humiliated, and underrepresented in the mainstream media. When certain groups are ignored and stereotypically represented in the media, they are symbolically annihilated. Early researchers introduced this concept in the 1970s, and it has been used in many studies. In recent history, several disadvantaged and historically marginalized groups have been symbolically annihilated by the media [20]. In such cases, social value of those groups declines, they are excluded from the media, and their problems are trivialized. “Near-total absence” gives an implicit message about the members of social groups who are excluded from the media [21]. This causes lack of knowledge about underrepresented groups since peculiarities, and implied cultural value of the minority groups are learnt through media consumption. Since media exposure affects beliefs, attitudes, and behaviors, when a social group is ignored in the media, public assumes that the group is nonexistent, insignificant, or worthless [22].

The role of the media has been mostly analyzed in terms of disseminating accurate information, and the use of social media for easy access to information on healthcare during the COVID-19 pandemic. Media framing of the COVID-19 pandemic on the leading media outfits of the United States of America and Europe was also indicated by researchers. “Economic consequences” of the COVID-19 on the individuals, organizations, or the country were preponderant frame [23]. Another research showed minorities and migrants have faced difficulties to access accurate information on COVID-19 due to language and cultural barriers. They used social media for advice that may be incorrect [24].

In this study, first, the role of the media during pandemics/epidemics will be discussed. Second, representation of Syrian refugees in Turkish news media will be exhibited. Third, the assistance for Syrian refugees since their access to Turkey will be delved into. Fourth, the research on the representation of Syrian refugees in Turkish media during the pandemic will be presented in detail. Lastly, symbolic annihilation of Syrian refugees in Turkish news media will be elaborated.

## Background

News media play a key role during public health crises since news media attract public attention; affect attitudes, and public opinion. Media coverages affect social representations of emerging infectious diseases by producing information during pandemics/epidemics. When Ebola (the first outbreak of the Ebola epidemic was in Ebola River in Democratic Congo Republic in 1976) spread across other parts of Africa, it received widespread media coverage in the British press in the mid-1990s. Especially in the British tabloids, Ebola was depicted as intrinsically African and spread partly by African traditions of close contact with monkeys as well as eating them ([25] p966). The SARS epidemic, severe acute respiratory syndrome, which occurred in Hong Kong, took media attention in the spring of 2003. News stories on SARS pervasively covered the themes of difference between the West and the East through othering Chinese authorities and Chinese people’s dirt, unhygienic lifestyles in the British press ([26] p2568). When the H1N1 influenza emerged in Mexico in June 2009, the WHO declared a pandemic. However, media stories which pointed out that the outbreak supposedly started in Mexican pig farms played a role in the denunciation of Mexicans and Latinos as usual suspects in the US in 2009 ([27] p1), while also exonerating the dominance of white Americans.

The media had an enormous influence on public during the SARS epidemic. It was shown that consistent messages about preventive measures, mask use and hygiene practices on television and in the newspapers affected the total spread of the germs. Rise in media reports and encouraging news stories on SARS and H1N1 epidemics impacted social distance practices, vaccination behaviors, severity, and length of the epidemic/pandemic ([28] p1–2-10, [29] p870). Media exposure served to enhance H1N1 knowledge of people during the H1N1 pandemic in 2009; the degree of neighborhood social capital, community trust, social networking and social bonds enhanced information flow and affected positive health behaviors including parents’ decisions on vaccination ([30] p4860–5).

A study shows that media coverage cannot be responsible for the control of transmission of the epidemic by itself, but it helps shorten the time of the secondary peak by alerting and making aware of the public to the virus ([31] p50). Regarding this, media have a considerable effect on the pattern and the scale of transmission ([32] p163). When media guide the public, it can mitigate emerging infections during the early stages of an outbreak ([33] p9). The effects of the media coverage become more significant when visibility of issue increases and when news stories are consistently biased in one direction ([34] p15).

### Syrian refugees in Turkish news media

Turkish news media and Turkish state officials hardly ever mentioned Syrian refugees since the outbreak of the pandemic although Syrian refugees have been stereotypically represented in Turkish news media for years. Prevailing perceptions of Syrian refugees were mostly affirmative in the early years of the Syrian refugee crisis. General social acceptance of Turkish society towards Syrian refugees was remarkable between the years 2011 and 2014. The descriptions of Syrian refugees as “people who escaped from persecution and tyranny”, “our guests in Turkey” and “our brothers and sisters of the same religion” were examples of the positive attitudes. However, there were also some negative perceptions such as “people who are a burden on us” or “parasites/beggars” ([35] p66–67).

Syrian refugees were depicted negatively since their arrival in Turkey by news media. Discriminatory framings in national press between 2011 and 2014 helped justify backlash of “threat”, economic burden, and security problems without regarding human rights perspective ([36] p157–77). Negative media representations in local and national dailies between 2013 and 2015 affected adversely social integration of Syrian refugee women ([37] p15–8). Their vulnerabilities and poverty were partly mentioned in the news media along with depictions such as criminals, fugitives, and as a burden on national economy in 2014 ([35] p78–79). Syrian refugees became the target of hate speech for the first time in 2015 in Turkish press ([38] p8,12). They were exposed to hate speech pervasively in the press and online ([39] p400–1).

They were characterized as a potential source of public disturbance, represented as a burden for Turkish economy, and portrayed as a threat against Turkish family structure and social order in the press in 2016 ([40] p8–9). News media differently covered Syrian refugees in the national and local context. National press dramatized problems of refugees such as health problems, deaths, and impoverishment more than local press. Only 11.3% of news stories in 2017 and 2018 covered education, healthcare, and housing issues of Syrian refugees ([41] p33). Syrian refugees were depicted as a burden on Turkish public services, claiming that Syrians had priority over Turks in health access, business ownership and other governmental resources ([42] p383). Newspaper articles expanded otherization of Syrian refugees in the society ([43] p46). Syrian refugees were the most targeted group of hate speech in the Turkish press in 2019 [44]. Online hate speech against Syrian refugees was associated with political and economic problems, division in terms of Islam and secularism, hate against government policies. Online hate speech posed a more severe threat against Syrian refugees more than traditional media. Online hate speech reproduced pre-existing otherization of Syrian refugees and spread the discourse of discrimination. This affected very much ordinary individuals compared to other individuals with extreme beliefs. ([45] p147–8, p153). Hate, hostility, and racist discourses on social media, involved negative depictions which intensified ‘banal nationalism’ ([46] p9).

Hegemony of dominant groups and their media access bring out stereotypical media representations of minorities in the interest of majority groups. Superiority of dominant groups is strengthened by social representations of outgroups and stereotype content in the media ([47] p402–3, p410). Stigmatized groups face discrimination, which encompasses structural discrimination, too. Structural discrimination occurs when institutional policies deprive stigmatized groups ([10] p372– 3[48]; p1527). Stigma brings “resource-reducing discrimination” which causes a variety of problems such as unemployment, housing, education, and access to healthcare ([49] p814) Stigma processes affect the distribution of life chances including medical care [50].

### “Support” for Syrian refugees

Turkish state and several nongovernmental organizations provided some resources to meet the needs of refugees since the displacement of refugees following the outbreak of Syrian crisis in 2011 [51]. General Directorate of Migration Management and the Migration Board were established in 2013 to implement government policies and strategies aimed at supporting migrants and refugees. After Syrians flee to Europe, the EU considered a crisis when over one million Syrian refugees arrived in Europe in 2015. Following all member states could not reach a solution of receiving refugees fairly within the EU borders, cooperation with the origin and transit countries came to the fore. Since the EU needed an urgent solution to stop refugees crossing the borders, the EU regarded Turkey as a potential partner ([52] p2–3). The EU and Turkey agreed on Joint Action Plan in October 2015 cooperating on preventing undocumented migration to the EU borders [53]. Following the Joint Action Plan, the EU-Turkey Statement of November 2015 was agreed on and according to the Statement, the EU committed an initial 3 billion euros to Turkey to be spent for improving the socio-economic situation of the Syrians under temporary protection. Through ‘structured and more frequent high-level dialogue’ between the EU and Turkey, the EU pledged to accelerate visa liberalization once the requirements of the Roadmap are met. In return to concessions of visa-free travel for Turkish citizens in the Schengen zone, re-energizing accession negotiations of Turkey’s membership to the EU and opening of the new chapters, Turkey committed to prevent undocumented migration flows to the EU [54].

The EU-Turkey Statement of March 2016 agreed on to end the undocumented migration from Turkey to the EU in line with the Joint Action Plan. The EU committed to mobilize an additional three 3 billion euros until the end of 2018 and to accelerate the visa liberalization roadmap aiming to lift the visa requirements for Turkish citizens until the end of June 2016. Turkey would take any necessary measures to prevent new undocumented migration and resettlement process of Syrians would be ensured according to the Statement. As of 20 March 2016, Turkey would readmit all new undocumented migrants crossing from Turkey to the Greek islands [55]. Since the EU needed Turkey to keep Syrians out of the EU borders and Erdogan threatened the EU to open the doors to Greece and Bulgaria in February 2016, the EU had to expand the concessions of Turkey [56]. Recognizing and treating Turkey as a safe country of return were among these concessions, which lead to ignoring human right violations and authoritarian crackdowns ([57] p328). Weaponizing Syrian refugees by using them as a tool to extract concessions from the EU helped Erdogan to be less subject to the EU’s scrutiny and criticism ([58] p178). Empowering the Erdogan regime has undermined Turkish democracy ([59] p207) and press freedom. International refugee law has been breached by Greece, the EU and Turkey in many aspects according to the expert [60]. Erdogan threatened to open the borders to send back Syrian refugees, and when he opened the northern border with Greece in spring 2020, asylum seekers were subject to violations of human rights on both sides of the border. At least one Syrian lost his life, while trying to enter Greece. Although illegal and violent pushback took place, the EU has not taken any action to protect international law in terms of protecting human rights and stopping pushbacks at the borders. The EU aimed at preventing enters of asylum seekers to its borders by providing “cooperation packages” with third states and amended the *New Pact on Migration and Asylum* in September 2020. It has been considered a further step in reducing access to asylum in the EU and increasing deportations from EU territory [61].

Before the outbreak of the pandemic, according to Turkey’s Sustainable Development Goal (SDG) Voluntary National Review report in 2019, significant progress was achieved especially in social policies including reducing poverty, inequalities and accessing to basic services and health care. In this regard, registered Syrian refugees who hold a temporary ID number may receive all health care, free of charge. Unregistered Syrian refugees who do not have a temporary ID number are only provided with limited services and all Syrians are entitled to go directly to the health care centers of the Ministry of Health ([53] p46). Within the framework of the government policy, Migrant Health Centers (MHCs) were established in densely populated areas by Syrian refugees aiming at breaking language and cultural barriers as stated in the government report ([62] p46). However, prominent difficulties of Syrian refugees in access to basic services, registration issues, integration problems, stigmatization, discrimination, low socioeconomic level, and a general decline in the quality of life persist ([63] p5–8).

Syrian refugees have been extremely at risk due to the poor living conditions in crowded houses, insufficient sanitation conditions and lack of income since the outbreak of the pandemic ([63] p3–8). The pandemic exacerbated the existing problems in many areas, particularly access to healthcare since Syrian refugees are not entitled to healthcare in the provinces other than they reside ([64] p 7[65]; p31). Moreover, Arabic-Turkish language barrier and lack of translators in health care facilities in most places remain ([66] p1439), which result in treatment gap ([67] p2). Although some health measurements were taken in 178 Migrant Health Centers in 29 provinces of Turkey ([68] p9–10), according to the report of Refugee Support Association, Syrian refugees were reluctant to go to health centers due to problems of social integration, mainly such as language barrier, worries, and lack of information. It was revealed that even employed Syrian refugees were reluctant to go to hospitals due to fear of being deported, eviction or dismissal, in case of testing positive. According to the survey, 52% of Syrian refugees did not have sufficient information on updates on access to healthcare, such as hospital appointments, medicines, and renewal of health reports. ([69] p25–6]).

Syrian refugees have become more impoverished during COVID-19 pandemic [70]. A survey revealed that before the pandemic, unemployment rate was only 18% among the refugees, but then it increased to 88% after March 2020. Participants (43%) stated that they lost their jobs because the company or institution they work for stopped their activities. Dismissal (18%) and not being able to find a job (12%) were among the causes of unemployment. Syrian refugees had inadequate access to food (63%) and hygiene (53%) as of March 2020. They had difficulties in paying their rents, bills, and meeting basic needs due to increased expenditures (90%). 48% of children enrolled in the schools could not benefit from distance education ([70] p21–3).

A survey was conducted in 12 provinces of Turkey to reveal the impact of COVID-19 pandemic on employment of Turkish citizens and Syrian refugees, in May 2020. Results indicated that Syrian employees became more fragile than Turkish employees and women suffered the most. Loss of income was 88% for Syrian refugees, but it was only 50% for Turkish citizens. The rate of dismissal and unpaid leave of Syrians was higher than Turkish citizens. Nearly half of Syrian refugees lost their livelihood for an indefinite length of time. It was revealed that most of the refugees (90%) could not benefit from the COVID-19 support [71]. Content analysis of Turkish news media will demonstrate underrepresentation of the plight of the refugees in the research period.

## Method

In this research, the representation of the Syrian refugees in Turkish news media was analyzed through content analysis method. Content analysis is a research technique which includes selecting and collecting data, creating categories, coding rules, conducting coding, analyzing the findings, and presenting a conclusion. Content analysis is defined as breaking texts into categories based on a coding frame [72]. Content analysis is widely used in health and social research. It is applied for drawing a conclusion by systematically and objectively determining characteristics of messages. Researchers conduct content analysis to quantify and find meaningful connections, make inferences, create comparable amounts to distinctive features of messages in qualitative data such as texts. Content analysis includes both qualitative and quantitative research.

### Sampling

Through the analysis of news media content, I have sought to reveal representation of Syrian refugees in Turkish media. The data used in this research were provided by Media Monitoring Center.^4^ Purposive sample was selected between March 11 (the first confirmed case of COVID-19 in Turkey) and August 20 (the time of this research) to analyze news mentioning Syrian refugees. Mostly watched television evening (prime) news, the most widely circulated (over 100,000) newspapers, and their websites (first five of the most visited) were evaluated. Television evening (prime) news of *FOX TV*, *CNN Türk*, *TRT News* (public broadcaster), *Habertürk TV*, *Kanal D*, *Show TV*, *NTV* and *Star TV* were chosen in order of ratings in line with the data of the Reuters Institute’s Digital News Reports. The news stories from the newspapers *Sabah*, *Sözcü*, *Hürriyet*, *Posta*, *Türkiye Gazetesi*, *Milliyet*, *Akşam*, *Yeni Şafak* were analyzed in order of circulation in line with the data of Media Monitoring Center. News websites of *CNN Türk*, *NTV*, *Hürriyet*, *TRT News* and *Sözcü* were analyzed.

### Categorization

News stories mentioning Syrian refugees were analyzed through the categories which were created for the research question in this study: How many times and how were Syrian refugees represented in Turkish news media between March 11 and August 20, 2020? Four categories are determined: ‘Access to healthcare during the pandemic’ category includes news themes of prevention, and treatment of the COVID-19 disease. ‘Portrayal as a source of problem’ category includes negative depictions of Syrian refugees such as carriers of the disease, threat to Turkish population, burden on national economy. ‘Socio-economic issues and/or the smuggling of refugees’ category includes issues such as poverty, employment, education. The support Syrian refugees receive is mentioned in few of these news stories. In this category, the smuggling of refugees involves facilitation of Syrian refugees’ illegal entry into European states. ‘Acclaim for Turkey’s policies and/or criticism of the EU, the West & opposition parties’ category includes statements of government officials, power and success of Turkey in dealing with refugees, disapproval of the EU and mostly Greece. This category includes both acclaim for government and the criticism of the European countries and opposition parties because these topics were mostly covered in the same news story. Based on these categories, news stories were counted, presented in tables, interpreted, and the results were reported.

## Results

### Television news (Table 1)

**Table 1:** Number of television evening (prime) news stories between March 11 and August 20, 2020

	Evening (prime) news stories about Syrian refugees in Turkey				
Name of the TV Channel	Access to healthcare during the pandemic	Portrayal as a source of problem	Socio-economic issues and/or the smuggling of refugees	Acclaim for Turkey’s policies and/or Criticism of the EU, the West & opposition parties	TOTAL
Fox TV	–	–	–	–	0
CNN Türk	–	1	1	4	6
TRT News	–	–	–	9	9
Habertürk TV	–	–	2	4	6
Kanal D	–	1	–	1	2
Show TV	–	–	–	2	2
NTV	–	–	1	6	7
ATV	–	–	–	1	1
Star TV	–	–	–	1	1
A Haber	–	–	–	1	1
**TOTAL**	**0**	**2**	**4**	**29**	**35**

TV news barely aired Syrian refugees during the 163 days covered in this study. Most of the Turkish people identify television as their main and the most trusted source of news [73]. TV news predominantly aired news stories about Turkey’s policies on Syrian refugees and criticized immigration policies of the European countries. News stories about Syrian refugees just contained the voices and opinions of political actors without any criticism or different perspectives. News channels featured statements of the ministers and the president with a great emphasis to the “generosity” of Turkey. The minister of interior stated that there were no risks of COVID-19 infection within Syrian refugees living in the camps, thanks to the Turkish state who managed to take every precaution to protect them. Health minister stated there were no extraordinary rates of COVID-19 cases. However, no voices of Syrian refugees appeared in the news.

*Fox TV* news is the most trusted evening (prime) news with the highest rating, but unexpectedly, it did not mention Syrian refugees at all. In one of the news stories on *Kanal D,* the police found an illegal health center, delivery room, and pharmacy run by Syrian refugees. It was emphasized that these facilities could pose a risk to public health. Syrian refugees were portrayed as the direct cause of unhealthy conditions, and they were blamed for ignoring basic hygiene during the pandemic. There were no remarks regarding why refugees had to consult medical care in these facilities.

### Newspapers (Table 2)

**Table 2:** Number of news stories in newspapers between March 11 and August 20, 2020

	News stories about Syrian refugees in Turkey				TOTAL
Name of the newspaper	Access to healthcare during the pandemic	Portrayal as a source of problem	Socio-economic issues and/or smuggling of refugees	Acclaim for Turkey’s policies and/or Criticism of the EU, the West & opposition parties	
Sabah	–	–	4	22	26
Sözcü	2	6	2	1	11
Hürriyet	1	2	7	11	21
Posta	–	–	2	1	3
Türkiye Gazetesi	2	1	3	13	19
Milliyet	–	4	9	15	
Akşam	–	–	–	12	12
Yeni Şafak	1	–	2	12	15
**TOTAL**	**6**	**13**	**29**	**87**	**135**

News stories were overwhelmingly about the Turkey’s “successful” policies on Syrian refugees and acclaim for Turkish authorities. Immigration policies of EU, the West, and the opposition parties were criticized. It was claimed that the EU failed to keep its word on every aspect of refugee crisis. Moreover, it was implied that European countries were guilty of not stopping the alleged trafficking of children. Spending over “40 billion dollars” for refugees was repeated in several news stories, but news stories hardly ever covered access to healthcare.

*Sözcü*, as the only anti-government newspaper in this research, criticized the large population of Syrian refugees. Syrian refugees were portrayed as a threat, source of problem, and cause for the spread of pandemic. The lack of information on COVID-19 related issues of Syrian refugees were underlined. In an interview in *Sözcü*, journalists claimed that asking questions to the government about COVID-19 and Syrian refugees was almost constrained. *Sözcü* claimed that the real population of Syrian refugees was over 5 million.

News stories ignored the plight of Syrian refugees and socio-economic issues in general. However, socialist newspapers focused on socio-economic issues of Syrian refugees. One of them was *Birgün*, which published a few news stories mentioning problems such as lack of medical care, poverty, negligence, educational problems, and poor working conditions. The other socialist newspaper *Evrensel* wrote that they tried to get information about refugees from the government institutions. However, they stated that they were either not answered or political authorities said that they did not have any information.

### News websites (Table 3)

**Table 3:** Number of online news stories between March 11 and August 20, 2020

	Online News stories about Syrian refugees in Turkey				
Name of the news platform	Access to healthcare during the pandemic	Portrayal as a source of problem	Socio-economic situation and/or smuggling of refugees	Acclaim for Turkey’s policies and/or Criticism of the EU, the West & opposition parties	TOTAL
CNN Türk online	–	–	6	19	25
NTV online	–	–	11	8	19
Hürriyet online	1	2	21	24	48
TRT News online	–	–	9	16	25
Sözcü online	3	8	5	10	26
**TOTAL**	**4**	**10**	**52**	**77**	**143**

The websites *CNN Türk online, NTV online, Hürriyet online* and *TRT News online* mostly covered stories acclaiming for Turkish government like newspaper or television news stories. Turkey’s demands of financial aid from the EU frequently appeared in the news stories. Greece and other the EU countries were blamed for not helping Syrian refugees. *Sözcü online* criticized Turkish government for not helping Turks but spending resources on Syrian refugees. *Sözcü* asked questions about the precautionary measures to prevent the spread of Coronavirus among the refugees, and it claimed that refugees were among the causes of economic crisis. Online news stories hardly ever covered access to healthcare, too.

## Discussion

This study provided insights into Turkey’s news media coverage of Syrian refugees during the COVID-19 pandemic. News stories mentioning Syrian refugees and the impact of the COVID-19 on them were analyzed on television evening (prime) news, the most widely circulated newspapers, and the websites of them. The results indicated that Turkish news media predominantly ignored Syrian refugees, rendered them invisible and voiceless. The most common themes appeared in the newspapers were the “generous” support of the Turkish government for Syrian refugees and criticism of the EU policies towards refugees. The most watched television evening (prime) news hardly broadcast news stories about Syrian refugees although television remains the most important news source across Turkey. Invisibility in the media and exclusion of their voices induced symbolic annihilation of Syrian refugees. Lack of representation of Syrian refugees and ignoring vulnerabilities of them perpetuated the dominance of official viewpoints and political actors during the pandemic. Syrian refugees faced greater socioeconomic problems, issues in healthcare, discrimination, and attacks; but ignoring the plight of the refugees caused reinforcement of already existing inequalities. Contrary to some news stories; other news stories, surveys, and research reports revealed lack of hygiene measures and facemask shortage. Impoverishment and rise in discrimination against Syrian refugees were also some issues exposed in those studies.

Media representations affect world views and when a particular group is valued, the group is more visible in the media. Other groups and the whole society can find out more about their implied attributes and implied value of this group by media consumption [22]. Underrepresentation of Syrian refugees in Turkish news media demonstrated their low value in society. Moreover, epidemics reveal the valued social groups and increase stigma against disadvantaged groups such as racial minorities or refugees. Stigma reduces health-seeking, help-seeking, and treatment-seeking acts [74]. The findings in this research corroborates stigmatization of disadvantaged groups during pandemics. Media representations can partly determine public opinion and change attitudes towards certain groups. Regarding this, rise in intolerance for Syrian refugees and backlash in Turkish society can be associated with less coverage of the plight of Syrian refugees as they have been stigmatized as economic burden to the welfare system of Turkey. Moreover, regarding widespread negative attitudes against Syrian refugees in the urban population as one of the most prominent reason for Justice and Development Party’s historic loss in local elections in 2019, should be taken into consideration. Symbolic annihilation of Syrian refugees during the pandemic has served the authoritarian regime by obscuring the plight of Syrian refugees. In authoritarian regimes, stigmatized groups like refugees may be more at risk of suffering from not only political or legal problems, but also from access to healthcare especially during the pandemics.

Symbolic annihilation causes beliefs about loss of importance of needs and rights of stigmatized groups such as refugees. Lack of information adversely affects public behavior and public health. Ambiguity with an invisible threat such as a virus may induce the spread of misinformation [75]. The results of this study support shortcomings of information on Syrian refugees and their access to healthcare. Most of the news stories were about Turkey’s “political achievements” and Turkish government rather than Syrian refugees. Analysis of news representation showed that Syrian refugees were virtually non-existent which is defined as symbolically annihilation.

The findings reveal the authoritarian pressure on news media and lack of media pluralism in Turkey since most of the news stories focused on acclaim for authoritarian Justice and Development Party (AKP), namely Erdogan. The critical role of news media is to serve democracy by encouraging public debate on all issues, not bolstering dominant perspectives without any criticism which will not help inclusion of the refugees in Turkish society or reinforce refugee integration. Media’s indispensable role in democracies is to represent refugees and all disadvantaged groups fairly.

## Conclusion

The current study demonstrated that Syrian refugees were symbolically annihilated by Turkish news media in the research period during the pandemic. It was indicated that the concealment of information and symbolic annihilation of disadvantaged groups could potentially cause health disparities and irreparable harm to public health. Moreover, inequities exacerbate when predicaments of stigmatized groups are ignored in the news media. However, accurate and vital information on pandemics and stigmatized groups such as refugees can increase access to healthcare and reduce the spread of infectious diseases globally. Media can promote health equity through disseminating more stories about health disparities and representing all disadvantaged groups.

## Data Availability

The datasets used and/or analyzed during the current study are available from the author on reasonable request.

## Footnotes

1 On March 22, Syrian officials announced the first confirmed case of COVID-19.

2 According to the 1951 Refugee Convention, a refugee is someone “owing to well-founded fear of being persecuted for reasons of race, religion, nationality, membership of a particular social group or political opinion, is outside the country of his nationality and is unable or, owing to such fear, is unwilling to avail himself of the protection of that country; or who, not having a nationality and being outside the country of his former habitual residence, is unable or, owing to such fear, is unwilling to return to it*.*” The 1951 Convention had geographic and temporal limitations. Only persons fleeing events occurring before 1 January 1951 within Europe were considered refugee within this limitation. The 1967 Protocol removed the limitations. However, the Republic of Turkey holds the geographical limitation to the 1951 Refugee Convention. Syrians are not provided refugee status in Turkey. Turkey adopted EU-inspired Law on Foreigners and International Protection (LFIP), which is a legal framework for asylum in Turkey in April 2013. It established Turkey’s obligations towards all persons in need of international protection, regardless of country of origin. Turkey adopted Temporary Protection Regulation for on October 22, 2014. Syrian nationals, stateless persons, and refugees from Syria are under temporary protection and they cannot be sent back to Syria according to this regulation with the rights. In this study, Syrians are called as refugee in terms of sociological definition. In this respect, people who flee political violence including threat for persecution are considered refugee regardless of legal framework of countries. For further information on definition of refugee, see [3] [4]. For further information on legal framework for Syrian refugees, see https://www.unhcr.org/tr/en/refugees-and-asylum-seekers-in-turkey

3 For further information on the legal refugee status and asylum seekers in Turkey, see also [6] [7].

4 Media Monitoring Center is based in İstanbul, Turkey. It was founded in İstanbul in 1999 and it has agencies in Germany and Azerbaijan, currently. It is an independent media company which works in the media monitoring and measurement. See https://medyatakip.com.tr/en/
